# Design and fabrication of a vigorous “cavitation-on-a-chip” device with a multiple microchannel configuration

**DOI:** 10.1038/s41378-021-00270-1

**Published:** 2021-06-02

**Authors:** Farzad Rokhsar Talabazar, Mohammad Jafarpour, Merve Zuvin, Hongjian Chen, Moein Talebian Gevari, Luis Guillermo Villanueva, Dmitry Grishenkov, Ali Koşar, Morteza Ghorbani

**Affiliations:** 1 Faculty of Engineering and Natural Science, Sabanci University, Tuzla, Istanbul Turkey; 2 Sabanci University Nanotechnology Research and Application Center, Tuzla, Istanbul Turkey; 3 Advanced NEMS Laboratory, École Polytechnique Fédérale de Lausanne (EPFL), CH-1015 Lausanne, Switzerland; 4 Department of Biomedical Engineering and Health Systems, KTH Royal Institute of Technology, SE-141 57 Stockholm, Sweden; 5 Division of Solid State Electronics, Department of Electrical Engineering, The Ångström Laboratory, Uppsala University, Uppsala, Sweden; 6 Center of Excellence for Functional Surfaces and Interfaces for Nano-Diagnostics (EFSUN), Sabanci University, Orhanli, Tuzla, Istanbul Turkey

**Keywords:** Engineering, Physics

## Abstract

Hydrodynamic cavitation is one of the major phase change phenomena and occurs with a sudden decrease in the local static pressure within a fluid. With the emergence of microelectromechanical systems (MEMS), high-speed microfluidic devices have attracted considerable attention and been implemented in many fields, including cavitation applications. In this study, a new generation of ‘cavitation-on-a-chip’ devices with eight parallel structured microchannels is proposed. This new device is designed with the motivation of decreasing the upstream pressure (input energy) required for facile hydrodynamic cavitation inception. Water and a poly(vinyl alcohol) (PVA) microbubble (MB) suspension are used as the working fluids. The results show that the cavitation inception upstream pressure can be reduced with the proposed device in comparison with previous studies with a single flow restrictive element. Furthermore, using PVA MBs further results in a reduction in the upstream pressure required for cavitation inception. In this new device, different cavitating flow patterns with various intensities can be observed at a constant cavitation number and fixed upstream pressure within the same device. Moreover, cavitating flows intensify faster in the proposed device for both water and the water–PVA MB suspension in comparison to previous studies. Due to these features, this next-generation ‘cavitation-on-a-chip’ device has a high potential for implementation in applications involving microfluidic/organ-on-a-chip devices, such as integrated drug release and tissue engineering.

## Introduction

Hydrodynamic cavitation (HC) is a phase change phenomenon involving a liquid and commences when the static pressure drops to a critical value—the saturation vapor pressure. This phenomenon includes a progressive vaporization cycle of the generation, growth, and implosion of bubbles. Small bubbles form in low-pressure zones, typically at the entrance of a flow restrictive element. Inertial cavitation bubbles grow in a successive cycle until they reach a high-pressure area, where they collapse. Cavitation is mostly known as an undesirable phenomenon, and most of the studies^1–3^ on the physics of cavitation aimed to prevent it or decrease its detrimental effects. However, applications of HC at the conventional scale have been successfully realized in the food and beverage industry^4–6^ and hydrometallurgy^7^. Furthermore, its extensive applications at the microscale are emerging, such as wastewater treatment^8^, biomedical applications^9–11^, energy harvesting^12,13^, and liquid phase exfoliation^14^.

HC has been extensively studied at the conventional scale during the last decade, with the construction of the related theory as well as practical design guidelines for cavitation. However, there is still a lack of knowledge on cavitation at the microscale. Due to the advantages of the use of microfluidic systems and many potential applications, it is inevitable that there will be an increasing need for new studies on and insights into the cavitation phenomenon at the microscale.

The design and fabrication of microfluidic devices capable of generating cavitation bubbles have become a critical subtopic in cavitation in small domains. Mishra et al.^15^ performed a pioneering study in the field. They employed semiconductor microfabrication techniques to fabricate microfluidic devices for investigating microscale cavitation. The devices housing a micro-orifice were fabricated on a silicon wafer and bonded to a Pyrex cover for flow visualization. In another study, Mishra and Peles fabricated microfluidic devices with a micro-Venturi to reveal the effect of the geometry on the generation of cavitation^16^. The results of these studies emphasized strong scaling effects on the physics of the microscale cavitation phenomenon. In comparison with the conventional scale, HC at the microscale exhibits different features. At a small scale, surface nuclei have an effective role such that the inception number is relatively small compared to the macroscale. Moreover, the cavitating flow patterns quickly transform into developed and supercavitating flow regimes, which is in contrast with macroscale studies^15^. Surface tension forces, the influential force related to surface nuclei, and the stream nuclei residence time are effective parameters in HC at the microscale.

Flow visualization is a vital tool for characterizing cavitating flows. It is therefore essential to consider the addition of a visualization capability while designing and fabricating cavitation-on-a-chip devices. Moreover, adding a visualization capability and being able to withstand high pressures at the same time constitutes a major challenge in microfabrication processes. To address this challenge, Medrano et al.^17^ fabricated double anodic-bounded Pyrex–silicon–Pyrex devices using microfabrication techniques. First, they bonded a thin silicon foil to a Pyrex cover and etched through the entire silicon layer to form microchannels. Then, the second Pyrex cap was bonded to the other side of the silicon. Their experimental results indicated that cavitation inception exhibited no difference for the devices with the Si–Pyrex and Pyrex–Si–Pyrex configurations. Qiu et al.^18^ presented a simple fabrication process to obtain microchannels with the capability to generate HC. They fabricated a microstep inside the Pyrex–Si channel using controlled wet etching instead of implementing the deep reactive ion etching (DRIE) method. Their fabricated microfluidic device enabled the formation of cavitating flows at high flow rates at the microscale.

Most of the studies on microscale cavitating flows involved laminar flows and low-pressure drops due to the limitations in the fabricated device^15–20^. Recently, Ghorbani et al.^21^ proposed a new generation of cavitation-on-a-chip devices, which had roughened sidewalls to facilitate cavitation inception and could withstand high pressures. In this context, they investigated the surface roughness effects on the cavitation phenomenon at the microscale. For this, a Si–Pyrex microfluidic device with surface roughness was fabricated by microelectromechanical system (MEMS) fabrication techniques. To obtain uniform roughness on the channel surface, the mass flow rate controller (MFC) method was applied in an optimized DRIE system before Pyrex bonding. They used the MFC to control the sulfur hexafluoride (SF_6_) gas flow rate to fabricate the specified nanograss structure on the channel surface. The resulting microfluidic devices were proposed to be a suitable platform for achieving cavitating flows under fully developed turbulent conditions at high Reynolds numbers and could withstand upstream pressures up to 900 Psi (6.2 MPa). In another study, Ghorbani et al.^22^ investigated the feasibility of utilizing the concept of “hydrodynamic cavitation-on-a-chip” in energy and biomedical applications. They studied the effect of poly(vinyl alcohol) (PVA) microbubbles (MBs) on cavitating flows and energy dissipation. They conducted experiments in three microdevices with different roughness elements, namely, structural sidewalls, roughened surfaces and smooth surfaces. Their results demonstrated that PVA MBs facilitated more facile cavitating flow generation compared to the pure water case. In a follow-up study, Ghorbani et al.^23^ investigated cavitating flows inside 24 sidewall roughened and 5 surface roughened/plain microfluidic devices. They concluded that the sidewall roughness elements significantly affected cavitation inception and flow patterns. The best conditions for cavitation generation corresponded to the microfluidic device with short and small sidewall roughness. In another study, Ghorbani et al.^24^ assessed the potential of a new approach inside microfluidic devices with enhanced surfaces. They characterized the cavitation phenomenon for cellulose nanofiber-stabilized perfluorodroplets (PFC5). The microfluidic devices were modified by assembling various size silica nanoparticles. In this study, supercavitating flow patterns, which are recognized by elongation of the vapor cavity at low pressure such that the vapor cavity length becomes longer than the channel size or the core of the microchannel is filled with vapor, were observed at a very low upstream pressure (1.7 MPa) for the case of PFC5, which corresponded to cavitation inception for the pure water case. The results of this study highlighted that as the cellulose nanofibers (CNFs) were not damaged, the droplets could be regenerated, and the system energy could be stored and released on demand with a closed-loop system.

As mentioned above, the previous designs of cavitation-on-a-chip devices mostly consisted of an inlet area, a microchannel, and an extension chamber^15,19,25^. In recent studies, the lateral walls were equipped with engineered triangular roughness elements, which could trigger cavitation^21,23^ by presenting more nucleation sites for bubble generation, leading to high-intensity cavitating flows at a lower upstream pressure. Moreover, roughness elements decreased the pressure drop required for cavitation inception and the generation of developed and supercavitating flow regimes.

This study aims to determine the feasibility of the cavitation-on-a-chip concept for the generation of cavitating flows at lower upstream pressures and to explore the capability of the presented device to be utilized in microsystem applications. In this regard, a novel microfluidic device with eight short parallel microchannels (micro-orifices) is presented as a next-generation cavitation-on-a-chip device. The interactions among the flow restrictive elements promote cavitating flows such that facile cavitation inception and intense cavitating flows are viable at lower inlet pressures than in previous state-of-art studies. The effects of PVA MBs as a cavitation facilitator on cavitation inception and development are also evaluated. The substantial decreases in the upstream pressure corresponding to cavitation inception and the capability to provide different cavitating flow patterns of different intensities within a single device prove the proposed device’s high performance and potential in terms of energy input for cavitation inception and the intense cavitation needed for emerging applications.

## Materials and methods

### Design and configuration of the microfluidic device

**Fig. 1:**
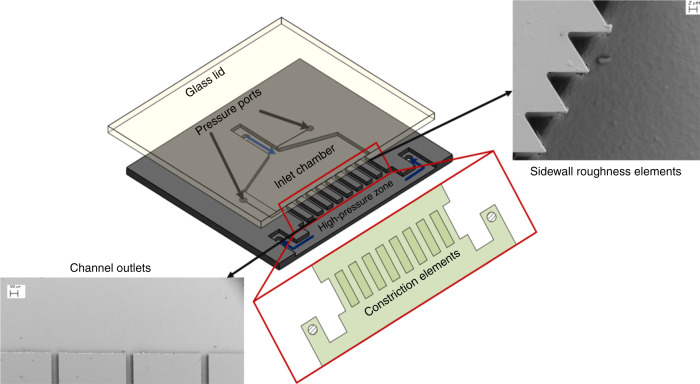
Schematic of the parallel multichannel arrangement (arranged in a cascade). **a** Overall configuration of the device, **b** sidewall roughness manifestation, and **c** outlets of the parallel microchannels

Figure 1 displays a representative microfluidic device with parallel flow restrictive elements (arranged in a cascade). As shown in Fig. 1, the microfluidic device consists of an inlet channel, where the fluid flow is guided into the inlet chamber. The inlet chamber is designed as a rather long section to let the transient chaotic flow disappear before the fluid enters the nozzle area. Based on our previous studies^13,23,26^ on microfluidic devices with a single micro-orifice, the inlet chamber was kept 2000 μm wide for each nozzle. Hence, in the present microfluidic device, a 2000 μm wide area is considered for each nozzle in the inlet chamber. After the inlet chamber, the streamlines face a sudden decrease in the cross-sectional area to facilitate the inception of HC. The hydraulic diameter of the nozzles is 66.6 μm. This number is chosen based on our previous study on different microfluidic devices with different nozzle widths^26^, in which three different single-channel microfluidic devices were fabricated and tested with water and PFC5 droplets. Since the early nucleation of HC and intense cavitating flows are favorable for researchers working on cavitation applications in the industry, the corresponding width is chosen in this study. The main goal of this design is to enhance the cavitating flow by providing the inception of a cavitating flow at lower upstream pressures and to have a fully developed, i.e., more powerful, two-phase flow in terms of the energy release for a microfluidic device with multiple restrictive elements. The results of the previous study show that the inception cavitation number for this device is significantly lower than that of the other geometries. In addition to the mentioned features, the sidewalls of the nozzle area are equipped with engineered roughness elements (as shown in Fig. 1b). There are two design parameters regarding roughness elements: the total length (*L*_R_) and height of the triangular elements (*H*_R_). The roughness elements are added to the sidewall to facilitate the formation of cavitating flows by changing the tensile strength of heterogeneously nucleated bubbles. The full theoretical analysis of the roughness element effect can be found in our previous study^27^. Finally, the streamlines enter a high-pressure zone, which could enhance the collapse of the bubbles generated in the nozzle area. The two-phase flow consisting of the intact bubbles and liquid phase is directed from the outlets (Fig. 1c) to the sides. As a result, the present design provides a negative static pressure area for the generation of HC bubbles as well as the reverse pressure gradient required for the collapse of bubbles. Table 1 exhibits the geometrical parameters of the fabricated devices.

**Table 1:** Geometrical characteristics of the devices.

Physical configuration	Range
Microchannel length (*L*)	1 mm
Microchannel hydraulic diameter (*D*_h_)	66.6 μm
Extended region length	2 mm
Extended region width	7800 μm
Length of the roughness elements (*L*_R_)	1/3*L*
Height of the roughness elements (*H*_R_)	0.1*D*_h_

### Fabrication procedure of the microfluidic device

**Fig. 2:**
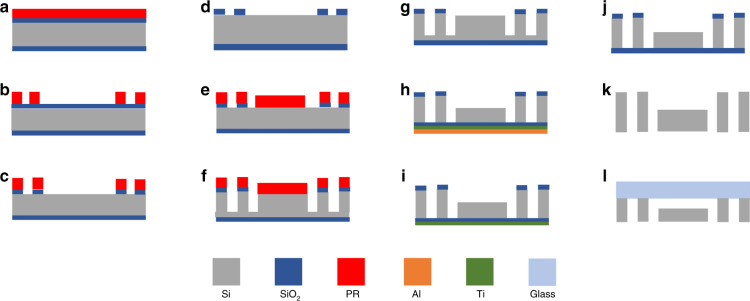
Fabrication process flow of the microfluidic device. **a** Photoresist casting on a silicon dioxide-coated wafer. **b** Maskless lithography for channel design. **c** SiO_2_ etching, **d** resist stripping, **e** second lithography to open the inlet–outlet and pressure ports. f DRIE for Si etching. **g** Photoresist stripping. **h** Ti and Al coating to protect the wafer as well as etching and second DRIE for etching through the wafer to open inlets, outlets, and pressure ports. **i** Wet etching of Al. **j** Wet etching of Ti. **k** Wet etching of SiO_2_. **l** Anodic bonding of the substrate to glass after etching the silicon dioxide layer completely.

Double-side polished 〈100〉 silicon wafers with a thickness of 525 µm were ground down to a thickness of 250 µm and coated with 500 nm-thick SiO_2_ layers on both sides (Fig. 2a). Four micron thick AZ 9221 photoresist (PR) was coated with RiteTrack88 after wafer priming with HMDS (hexamethyldisilazane) to enhance the adhesion onto the oxide surface (Fig. 2a). Direct writing lithography of the features drawn by the Layout Editor software was performed with a maskless lithography process (MLA150, Heidelberg). Each design took approximately 20 min to be written on the wafer (Fig. 2b). The patterned SiO_2_ layer was removed with an ICP-based high-density plasma source (SPTS APS) at 10 °C with a 3:1 SiO_2_:PR selectivity; it took ~3 min to completely etch the SiO_2_ layer (Fig. 2c), and the photoresist was removed (Fig. 2d). After the second step of photolithography (Fig. 2e), a DRIE (Alcatel AMS 200 SE) step was performed (Fig. 2f) to achieve silicon etching at 30 °C with a 75:1 Si/PR selectivity; 200 µm Si was etched after ~80 min, and the photoresist was stripped (Fig. 2g). At this point, 10 nm of Ti and 2 µm of Al were sputtered on the backside of the wafer using the Pfeiffer SPIDER 600 sputtering system to protect the wafer from breaking due to the stress generated by the DRIE process (Fig. 2h). Ti is the adhesive layer for Al adhesion to SiO_2_, and the Al coating without a Ti coating is flaky and inappropriate for the DRIE process since the backside of the wafer is rough and prevents electrostatic clamping. Inlets, outlets, and pressure ports were opened, and the success of the process was monitored by the laser intensity. When the laser intensity was constant, this suggested that Si was completely etched. The live image of the wafer provided a vibrating display when the wafer was closed at the end. After opening inlets, outlets, and pressure ports (Fig. 2h), wet etching of Al, Ti, and SiO_2_ layers was performed in an aluminum etcher (ANP H_3_PO_4_ 85% + CH_3_COOH 100% + HNO_3_ 70%, 300 nm/min, 35 °C), HF (1%, >1000 nm/min, 20 °C) and BHF, respectively (Fig. 2i–k). To remove all the organic residue, piranha cleaning was performed; baths of concentrated sulfuric acid (H_2_SO_4_ 96%) were heated to 100 °C for the process, hydrogen peroxide (H_2_O_2_ 30%) was then poured into them, and the wafers were dipped into the baths. The process was completed with rinsing and drying by a nitrogen gun. Spin/rinse/dry was not used because the wafers were thin, and this process might result in broken wafers. To be able to have a closed channel structure patterned Si wafers and Borofloat-33 glass wafers were anodically bonded (Süss SB6, vacuum anodic bonder) (Fig. 2l).

### Experimental setup and procedure

**Fig. 3:**
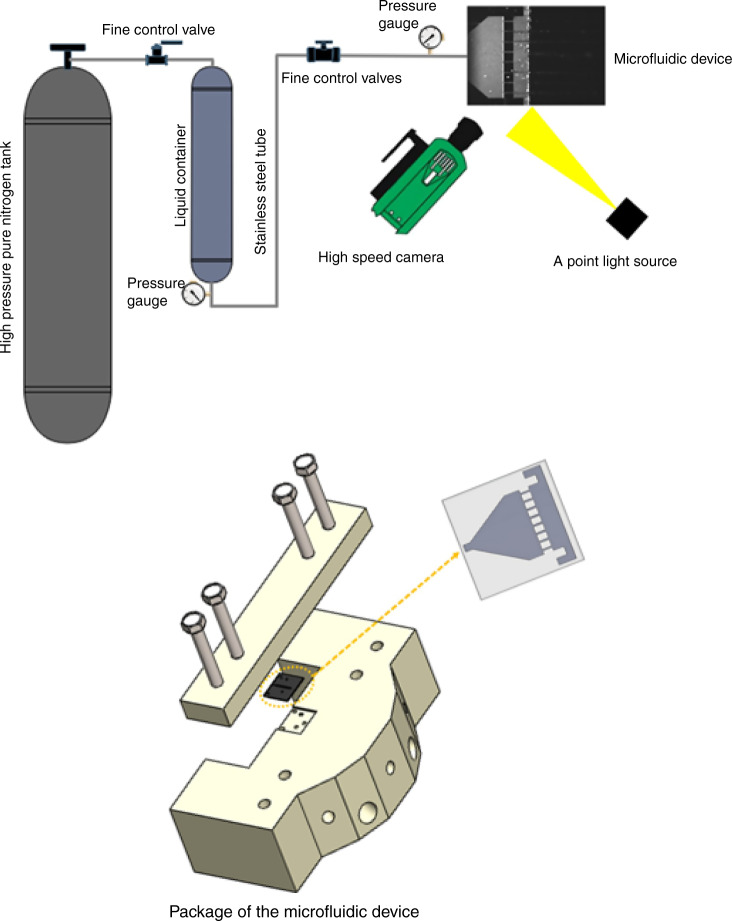
HC experimental setup. Visualization of cavitating flows and package used for securing and sealing the microfluidic device.

The desired inlet pressure was supplied to the system by a high-pressure nitrogen tank (Linde Gas, Gabze, Kocaeli, Turkey) from the top of a steel liquid container (Swagelok, Erbusco, Italy). The microfluidic device was sealed with the aid of an aluminum package with an appropriate inlet and outlet. The package was connected with fine valves and stainless steel (Swagelok, Erbusco, Italy) tubing to the fluid container. The experimental setup (Fig. 3) was equipped with pressure sensors (Omega, Manchester, UK, with an accuracy value of ±0.25% and a range of up to 3000 psi) to measure the upstream and downstream pressures. Micro O-rings and tight connections were used to avoid any leakage in the system. The glass wafer assisted in visualizing the fluid flow inside the channels. A double-shutter CMOS high-speed camera (Phantom VEO-710L) with a resolution of 1280 × 800 pixels and a pixel size of 0.02 mm, along with a macro-camera lens (type K2 DistaMax) with a focal length of 50 mm and an f-number of 1.2, was used to record cavitating flow patterns during the experiments. The package was installed at a distance of 200 mm from the camera to ensure that it was at the focal area, and only the central region of the lens was used for visualization. The images were acquired within very short time intervals at 12,200 fps with a shutter speed of 1000 s^−1^ and a 1 μs exposure time. Illumination in front of the microdevice was achieved by a point halogen light source to provide the background light required for better visualization with the high-speed camera.

The experiments were conducted for two working fluids (water and a PVA MB/water suspension with a mixture ratio of 1:19) by applying different inlet pressures from 0.2 to 1.1 MPa. The preparation of the PVA MBs was described in the study of Cavalieri et al.^28^. The PVA MB number in the suspension was evaluated using a Neubauer counting chamber (Bright-Line, Hausser Scientific, USA) and a transmitted light microscope. A MikroCam II (BRESSER GmbH, Germany) was used to take 16 images with 3000 × 4000 pixels at different locations of the chamber. The images obtained from the microscope imaging were analyzed by ImageJ software (version 1.53a, National Institutes of Health, USA) to determine the concentration and size distribution.

## Results and discussion

This study presents a new generation of cavitation-on-a-chip microfluidic devices with the facile generation of cavitation inside structured multiple parallel microchannels (cascade) configurations. For this purpose, two working fluids, water and a PVA MB–water suspension, were used as the working fluids. Cavitation inception and the cavitating flow morphology inside this microfluidic device were investigated for both working fluids.

### Demonstration of HC occurrence inside the multiple microchannel configuration

**Fig. 4:**
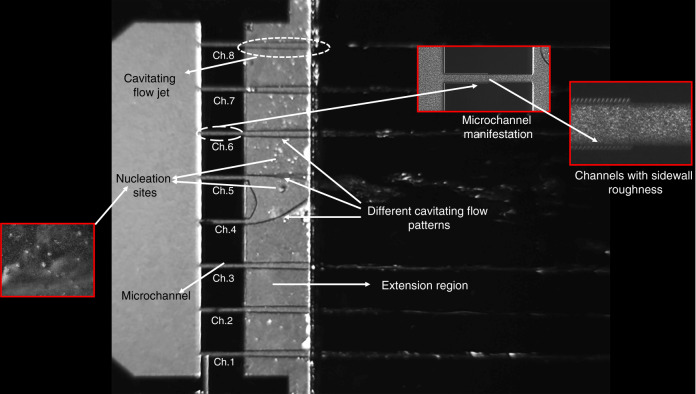
Overview of cavitation occurance. Different cavitating flow patterns have been formed inisde the multiple parallel microchannel configuration at *P*_i_ = 1.1 MPa.

Figure 4 shows cavitation bubble generation inside the microfluidic device, which has eight parallel microchannels with sidewall roughness elements. Cavitating flow patterns are visible in the extension region with emerging bubbles, which nucleate from the surface of the extended channel. This figure provides a comprehensive overview of emerging cavitating flows in this microfluidic device. As can be seen, various cavitating flow patterns are generated along the vertical axis of the extension region. Moreover, this overview clearly shows that cavitation occurs in all the parallel channels at an upstream pressure of 1.1 MPa. Accordingly, the general overview of the proposed microfluidic device suggests that it is possible to have different flow patterns at the same time, i.e., heterogeneous nucleation, bubbly flow, partial cavitating flow including sheet and cloud cavitation structures, and vortex formation. The aim of the presentation of different images of cavitation events in Fig. 4 is to emphasize that the proposed device has the capability to generate different patterns, which apparently have different levels of energy release. Therefore, this proof-of-concept study suggests that the proposed device could be an efficient multifunctional reactor, which can be exploited in applications where different levels of energy from bubble collapse (at the same time) are required.

**Fig. 5:**
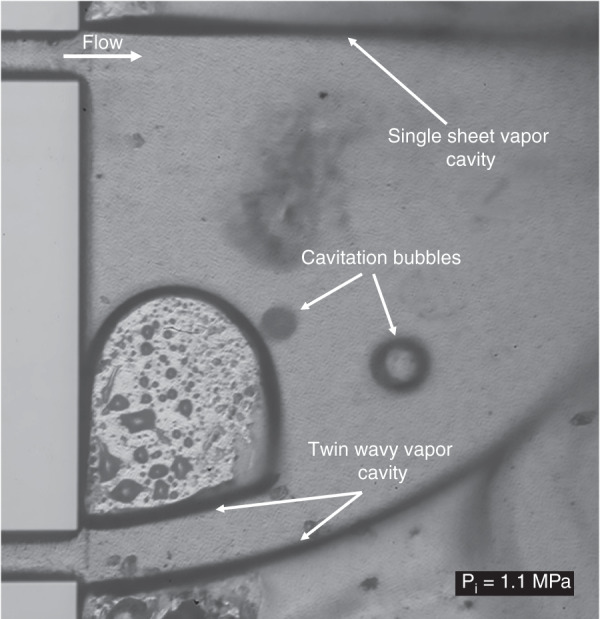
Different cavitating flow patterns P_i_ = 1.1 MPa.

Figure 5 displays the emerging cavitating flows at an upstream pressure of 1.1 MPa in the extension region downstream of two parallel channels (channels 4 and 5). Different cavitating flow patterns such as partial cavities, including sheet and cloud cavities, and single cavitation bubbles are apparent at a constant upstream pressure inside the multiple microchannel device configurations. As shown, a single sheet vapor cavity, a twin wavy cavity, and cavitation bubbles from the surface of the device are generated in the extended channel. According to the previous studies^8^, it is hard to see more than one cavitating flow pattern in unheated microfluidic devices with a single microchannel at a fixed upstream pressure, and a flow pattern shift can only be obtained by adjusting the upstream pressure or downstream pressure. In addition, as shown in Figs. 4 and 5, cavitating flows have different intensities in the extension region of each parallel channel, which implies that parallel microchannel configurations have the potential to provide cavitating flow patterns of various intensities at a fixed upstream pressure. In state-of-art cavitation-on-a-chip devices, however, cavitating flow patterns and their intensities are directly influenced by the upstream pressure (input energy), and there is a gradual shift from bubbly flow to fully developed cavitation^29^, which emphasizes the effect of the flow restrictive element configuration on cavitating flow patterns. Moreover, the pressures corresponding to cavitation inception (0.62 and 0.2 MPa for water and PVA MBs–water, respectively) in this proposed microfluidic device are lower than those in the state-of-art conventional cavitation-on-a-chip devices^21–23^ (1.65 and 2.34 MPa for water and 1.62 for PVA MBs–water). The device and orifice geometry has a significant role in determining the cavitation inception conditions and the intensity of cavitating flows. One of the geometrical parameters used to characterize cavitating flows is the ratio of the orifice area to the inlet area of the orifices (flow number)^30^. With a decrease in the flow number, cavitation occurs and develops earlier^31^. The proposed device was designed to have a smaller flow number parameter than conventional single orifice devices^21^.

Cavitation inception is an essential parameter in designing and fabricating a new generation of cavitation-on-a-chip devices. HC is a complex phenomenon with some critical issues in characterization. However, the cavitation number as a dimensionless number is widely used for characterizing the intensity of cavitation, which is defined as

1 $$\sigma = \frac{{P_{{\rm{{ref}}}} - P_{\rm{{v}}}}}{{\frac{1}{2}\rho _{\rm{{l}}}V_{{\rm{{ref}}}}^2}}$$

where *P*_ref_ is the reference pressure, which is the upstream pressure in this study and is measured exactly at the entrance of the cavitation device. The upstream pressure is the critical parameter in this study such that HC is generated by increasing this pressure while the downstream pressure is constant. *P*_v_ is the saturation vapor pressure of the liquid, *ρ*_l_ is the liquid density, and *V*_ref_ is the reference velocity, which is the average velocity inside the microchannels. *V*_ref_ is calculated by measuring the flow rate at the outlet and is represented as the cavitating flow average velocity (*V*_ref_ = *Q*/8*A*_p_) inside the microfluidic device. *Q* is the volumetric flow rate, and *A*_p_ is the microchannel cross-sectional area. It should be noted that the inception cavitation number (*σ*_i_) can be obtained by substituting *P*_ref_ with the pressure (*P*_i_) corresponding to the cavitation inception conditions.

Based on the experimental setup design and the locations of the measured parameters, such as the reference pressure and velocity, the cavitation number can be calculated using different approaches, which could lead to different ranges of cavitation numbers^32^. In this study, we describe the cavitation processes based on parameters measured from the mentioned open-loop experimental setup; thus, the upstream pressure is used as the reference pressure.

**Fig. 6:**
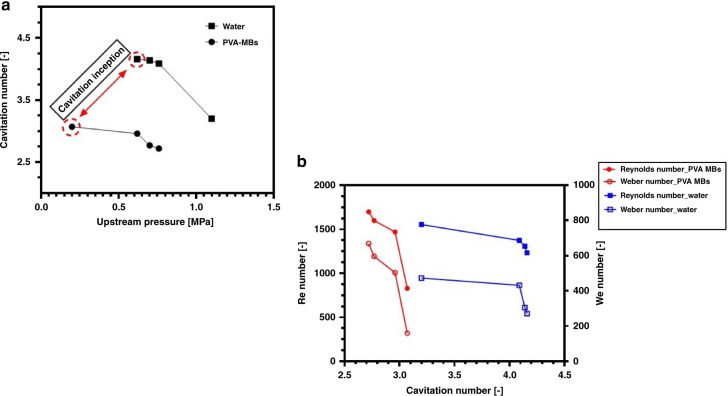
Cavitation number change according to the upstream pressure and nondimensional numbers. **a** Cavitation number with respect to the upstream pressure for water and PVA MBs. **b** Cavitation number variation with the Reynolds number and Weber number for the case of water and the PVA-MB suspension

Figure 6a shows the cavitation number with respect to the upstream pressure for the case of water and the PVA-MB suspension. Cavitation inception occurs at an upstream pressure of 0.62 MPa (90 Psi) with the formation of a cavitation cloud for water. Cavitating flows develop and intensify with increasing upstream pressure, and sheet cavitating flow can be obtained at an upstream pressure of 1.1 MPa (160 Psi). Accordingly, the upstream pressure required for cavitation inception in the proposed design is considerably lower than that in the single microchannel configuration in state-of-art cavitation-on-a-chip devices with the same hydraulic diameter (cavitation inception occurred at an upstream pressure of 1.65 MPa^21^). The results show that the cavitation inception number is 4.16 at 0.62 MPa and decreases to 4.14, 4.09, and 2.95 for upstream pressures of 0.68, 0.76, and 1.1 MPa, respectively.

As shown in Fig. 6a, cavitation inception occurs at a considerably lower upstream pressure for the PVA MB suspension than that in the water case. The inception pressure in the case of the PVA-MB suspension is 0.2 MPa (30 Psi), which is lower than those in the study of Mishra and Peles^15^ on cavitation in a single micro-orifice and our previous study^22^. The inception cavitation number, in this case, is 3.07 and decreases to 2.96, 2.77, and 2.72 for upstream pressures of 0.62, 0.68, and 0.76 MPa. Therefore, considering Fig. 6a, cavitation commences at lower pressure for the case of the PVA MB suspension, which means that lower input energy is required for bubble generation in this case. This is because PVA MBs provide more nucleation sites in the employed suspension. Furthermore, the cavitation number results show that the cavitation number for the PVA MB suspension decreases with a steeper slope than that for the pure water case in the upper-level range of upstream pressure (0.62–0.76 MPa), which suggests that the cavitating flow patterns in the case of PVA MBs develop more rapidly than those in the pure water case. The corresponding reduction in the inception pressure could be attributed to the microfluidic geometry design, surface and sidewall roughness element architecture, and earlier pressure recovery along the parallel microchannels.

**Fig. 7:**
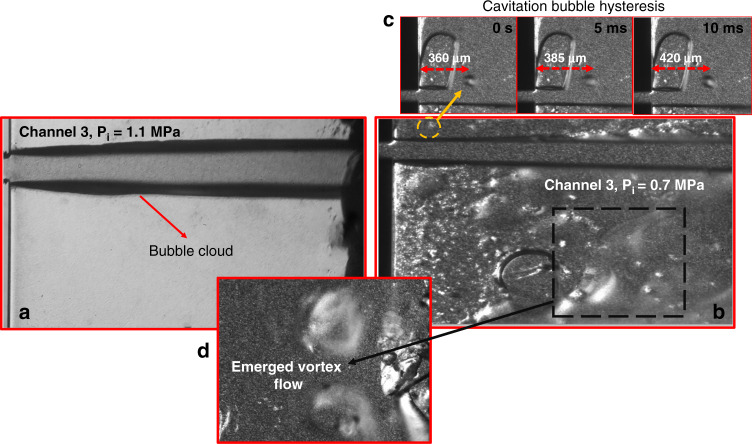
Cavitating flow patterns for the cases of water and PVA MBs. **a** Twin developed cavitating flow jet. **b** Twin sheet cavitating flow for the PVA MB suspension case, *P*_i_ = 0.68 MPa. **c** Cavitation bubble hysteresis in the case of PVA MBs, *P*_i_ = 0.68 MPa. **d** Microvortex flow generation in the extension region, *P*_i_ = 0.68 MPa.

**Fig. 8:**
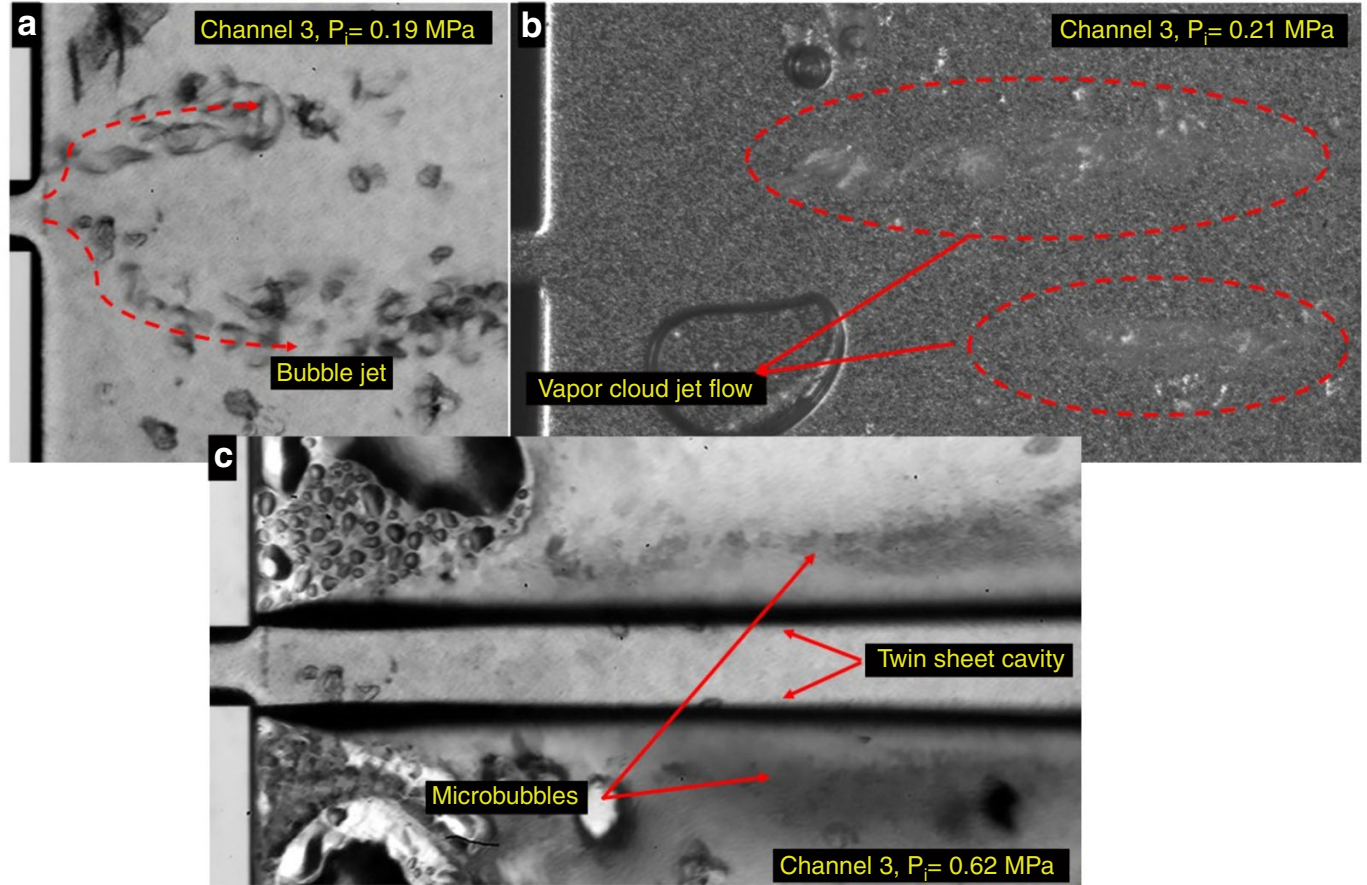
**Development of cavitating flow patterns in the case of the PVA-MB water.a** bubble jet P_i_ = 0.19 MPa. **b** vapor cloud jet P_i_ = 0.21 MPa. **c** twin sheetcavity P_i_ = 0.62 MPa.

The nondimensional Reynolds number (${\rm{{{Re}}}} = \rho v_{{\rm{{ref}}}}D_{\rm{{h}}}/\mu$; *ρ* and *μ* are the density and dynamic viscosity of the fluid, respectively, and *D*_h_ is the hydraulic diameter of the microchannels) is defined as the ratio of inertial forces to viscous forces. The Weber number (${\rm{{Wb}}} = \rho v_{{\rm{{ref}}}}^2D_{\rm{{h}}}/S$; *S* is the liquid surface tension) is the nondimensional number that represents the ratio of the viscous to surface tension forces. Figure 6b shows cavitation number variations with the Reynolds and Weber numbers. As seen in this figure, the Reynolds number corresponding to cavitation inception is lower for the case of the PVA MB suspension than for the pure water case. The bubble generation in the proposed configuration commences at a lower upstream pressure, and the corresponding Reynolds number accordingly has a lower value, as shown in Fig. 6b. Therefore, the flow velocity at inception is lower than that in our previous results^22^. The maximum Reynolds numbers in this study for both the water and PVA-MB suspension cases do not exceed 1698, implying that the flows inside the microchannels are laminar. According to the visualization results in Figs. 7a and 8c, with this proposed microfluidic device configuration, it is possible to attain developed sheet cavitating flow conditions at a lower Reynolds number under laminar flow conditions.

The Weber number can be used as a parameter to characterize the changes and deformation in the shapes and areas of bubbles. Accordingly, viscous forces dominate over the surface tension with an increase in the velocity. Therefore, the interfacial area of the two-phase flow increases with the Weber number due to the velocity increase, which leads to the development of cavitating flows. In this regard, as shown in Fig. 6b, the cavitation number decreases with the Weber number, implying that cavitating flow develops.

Figure 7 shows the twin sheet cavitating flow for the cases of water and the PVA MB suspension. A twin sheet flow pattern can be seen at an upstream pressure of 0.68 MPa for the case of the PVA MBs and at 1.1 MPa for the case of water. PVA MBs provide more nucleation sites for cavitation bubbles to grow and intensify cavitating flows. For this reason, the transition of the cavitating flow regimes is faster for the case of the PVA MB suspension than for water. Therefore, a twin sheet pattern can be seen at a lower upstream pressure. In addition, as shown in Fig. 7, there are different flow patterns in the extension region for the case of PVA MBs. Figure 7c displays the hysteresis phenomenon from the channel wall in the extension region before the twin sheet cavitating flow pattern emerges. There is a separation bubble before the formation of the sheet cavitating flow. This hysteresis phenomenon disappears within a short time due to the development of cavitating flow, which destroys the bubble separation. This finding agrees with the findings in a previous study^33^ on tip vortex cavitation. Figure 7d demonstrates a counterrotating vortex pair in the extension region around the cavitating flow. A circulation zone forms due to the breakoff in part of the sheet flow and transforms into a bubble cloud. The vortex pair emerges in the circulation zones, which are generated due to the cavitating flow momentum and have a direction opposite to the cavitating flow.

Figure 8 shows images of cavitating flows in the extension region of the cascade microfluidic device for the PVA MB suspension case. As seen in Fig. 8a, the emergence of a partial cavity is associated with cavitation inception at an upstream pressure of 0.19 MPa. The cavitating flow pattern transition from partial cavitating flow to the vapor cloud regime occurs at an upstream pressure of 0.21 MPa (Fig. 8b). An additional increase in the upstream pressure leads to the formation of an attached twin cavitating flow pattern at an upstream pressure of 0.62 MPa, as shown in Fig. 8c. These results highlight the fast evolution of cavitating flows for the case of the PVA MB suspension. In addition, as shown in Fig. 8c, bubble cloud formation occurs at the rear part of the sheet cavitating flow. The bubbles separate from the sheet regime and generate a bubble cloud in the circulation zone. In addition, the partial cavitation regime is apparent with the formation of cavitation near the channel in the extension region and then transforms into vapor cloud jet flow along the extension area at a farther distance. With an increase in the upstream pressure, sheet cavitating flow starts to emerge from the channel edge.

### HC effect on the bubble size inside the multiple microchannel configuration

**Fig. 9:**
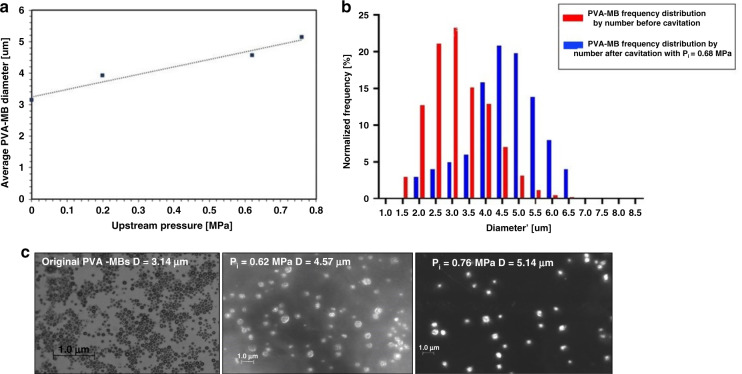
HC effect on PVA MB diameter. **a** PVA MB average diameter with respect to the upstream pressure. **b** PVA-MB frequency distribution by number at *P*_i_ = 0.68 MPa. **c** Microscopy images of the bubbles before and after the experiments.

Figure 9a displays the sizes of PVA MBs at different upstream pressures. As can be seen, the cavitating flow intensity has a substantial effect on the PVA-MB diameter size. The results show that the growth rate of MBs is faster for the developed cavitating flow condition compared to the cavitation inception condition, which implies that the microbubble size increases more at high upstream pressures. MBs continues to expand beyond a critical radius, where cavitation bubbles collapse. The previous studies^34^ on ultrasound cavitation reported that MBs reached a maximum expansion at a peak negative transmission pressure and then started to undergo compression immediately. Bubble fragmentation occurred both at the peak negative pressure and under compression. In HC, MBs expand with a sudden decrease in pressure. The dynamics of bubble growth and collapse in HC can be described according to the modified Rayleigh–Plesset equation^3^. The solution of this equation indicates that in the absence of thermal effects, a single bubble starts to grow in the low-pressure region until it reaches a critical radius. Therefore, the size of PVA MBs starts to increase when they enter a low-pressure region in the vena contracta along the microchannels.

In the proposed microfluidic device, MBs do not experience compression during HC. Therefore, cavitation inception and intensity are affected only by microbubble growth and fragmentation at the peak negative pressure. According to Fig. 9a, the diameter of PVA MBs at high upstream pressure is larger than that at a low upstream pressure, which implies that MBs experience a high peak negative pressure at a higher upstream pressure, and as expected, PVA MBs expand more according to previous studies on ultrasound contrast agents (UCAs)^34^ since MBs do not experience the compression cycle in HC as in the ultrasound case. In addition, the pressure decreases along the microchannels. Therefore, bubble expansion occurs gradually along the channels.

The other major parameter to be mentioned is the microbubble shell property. PVA MBs are MBs with polymer shells based on PVA, which provide sufficient stiffness to prevent gas bubble dissolution^35^. Moreover, the viscoelastic properties of PVA MBs have a significant role in stabilizing and restraining MBs at achieved diameters after HC. According to the results, the maximum expansion rate (ratio of the mean diameters of MBs after and before cavitation) of PVA MBs is 1.6 for 120 counted MBs for the same volume.

According to Fig. 9a, while the minimum local pressure during HC decreases linearly in the vena contracta region with the input energy (upstream pressure), the mean diameter of the PVA MBs increases with the same trend. PVA MBs are polymeric MBs that are much stiffer than air bubbles, and the behavior of these bubbles is dominated by the shell, not by the air inside them. The polymeric shell under the effects of pressure becomes curled and more flexible^36^, which leads to an increase in the size of MBs under a negative pressure gradient. This study shows that the PVA MB diameter changes almost linearly with increasing upstream pressure (accelerating the negative pressure gradient). In a previous study^37^ on AC, it was observed that the mean bubble size was dependent on the acoustic power and that the MB diameters increased almost linearly. This finding could be a good point to demonstrate a comparative study between effective parameters on HC (input power, velocity, pressure gradient, etc.) and AC (frequency, attenuation, etc.).

Figure 9b shows the normalized size distribution of PVA MBs for non-cavitating and cavitating flow conditions. A considerable change in the number of larger MBs occurs upon cavitation inception. More than 80 percent of the PVA MBs are larger than 4 μm upon cavitation inception, while only 20 percent of MBs have a diameter >4 μm under non-cavitating flow conditions. The bubble diameter frequency distribution indicates that the diameter of PVA MBs can be increased with exposure to HC. Most of the bubbles experience expansion in HC, and due to the low cavitation inception upstream pressure, PVA MBs cannot reach the critical radius under the cavitation inception conditions. For this reason, it can be claimed that the PVA MB size has a dominant role in the inception and intensification of the cavitation phenomenon by providing more nucleation sites for bubble growth.

## Conclusion

In this study, a new generation of ‘cavitation-on-a-chip’ devices is proposed. The device houses eight parallel structured short microchannels. This new design effectively decreases the upstream pressure required to initiate HC. Furthermore, the proposed microdevice allows the formation of different cavitating flow regimes at a constant upstream pressure at the same time, which is not achievable in state-of-the-art devices with a single flow restrictive element. The proposed device has the capability to provide cavitating flow patterns with the same intensity at a lower input energy. In addition, owing to the geometry of this device, the evolution of cavitating flow regimes is faster and more facile.

Two working fluids, water and a PVA MB suspension are used in the experiments. The motivation of utilizing PVA MBs is their potential in facilitating the generation of HC. The PVA MBs provide more nucleation sites, and inception can thus occur at a significantly lower upstream pressure in the case of PVA MBs compared to water. Moreover, emerging cavitating flows can develop faster. The PVA MB mean diameter increases during HC. The increase in the mean diameter of PVA MBs further facilitates the inception and evolution of cavitation by providing larger areas for bubble nucleation. Due to the many advantages it offers, the proposed ‘cavitation-on-a-chip’ device has a high potential for implementation in many applications involving microfluidic devices, such as integrated drug release and tissue engineering.

## Data Availability

The data that support the findings of this study are available from the corresponding author upon reasonable request.
